# Abstracts and Gleanings

**Published:** 1879-08-20

**Authors:** 


**On Codeia as a Sedative.**—No symptom is more distressing to a patient than frequent coughing, and none demands more judicious treatment on the part of the practitioner, if he would avoid undoing with his cough-mixtures all the good he is attempting by his more general therapeutic measures. In phthisis, the presence of anorexia makes us unwilling to give opium or morphia, and frequently, when we do so, we have reason to regret it. Many patients, especially gouty subjects, and those who suffer much from derangement of the liver, are intolerant of opium and morphia. On account of these difficulties, I have been led to employ codeia in such cases, in the hope that it might be of service, and it has succeeded beyond my anticipations. In phthisis it allays cough without disturbing the digestive system; and, in the other class of cases, I have found it tolerated when opium and morphia were not. As an instance of the latter, I may quote the case of a medical friend, a member of a gouty family, a frequent sufferer from migraine and derangement of the liver, and well aware of his intolerance of preparations of opium. He complained of a troublesome cough, depending on slight catarrh of the trachea and bronchi, and, at my suggestion, tried codeia, with all the benefit and none of the ill effects of opium. I prescribe the drug in doses of a grain, dissolved in syrup of tolu. The French medical papers constantly contain advertisements of codeia syrup, and probably it is well known as a cough tincture in this country; but I was not aware, and others may have been ignorant as I was, that it has those advantages over the preparations of opium and its other alkaloids. I therefore venture to call attention to it. Its value in diabetes is, of course, fully recognized.—*Brit. Med. Journal.*

### ABSTRACTS AND GLEANINGS.

**Dextro-Quinine**—By W. H. GALT, M.D., Louisville.—The desire to find a substitute for the sulphate of quinine among the cheaper salts of bark has been universally felt in all the regions where miasmatic diseases prevail. In accordance with this desire I have tried cinchonidia, cinchonia, quinoidine, and cinchoquinine. The results of their administration in some cases have been good, but in the long run have not been satisfactory so as to induce me to abandon the old, tried friend, the sulphate of quinine. In fact, I confess I could not get up the required faith in any other salt. It is said that the Tahitians are readily converted to the Christian faith, and seem to enter with zeal into the form of Christian worship. But when in great strait, "when they mean business," their prayers are all addressed to the old shark god of their fathers. No doubt my case is parallel with theirs, and I am slow to perceive the efficacy of any appeal except to my old shark, quinine.

I was much pleased with the tasteless preparation of cinchonidia with sugar of milk and bi-carbonate of soda. The first three cases in which I used it were cases of intermittent fever in children, in which the result was everything that could be desired. It was easily taken, and promptly subdued the paroxysm. In four following cases, however, I was disappointed in finding that the gastric irritation produced by it was so severe that I have not used it since. After this I concluded to make no more experiments with them.

Some months ago I received from Keasbey and Mattison two small vials of a new preparation from the bark, called *dextro*-quinine, because under the polariscope it gyrated to the right. What was the therapeutic value of this gyration, I did not see. It was an amorphous salt of reddish-brown color, totally unlike any of the alkaloids with which I was familiar, and I made up my mind that I would try it. In a day or two afterward I had a slight chill followed by a pretty severe fever, which lasted several hours, and passed off with a profuse perspiration. As quinine always affects me most disagreeably, I determined to try the new salt in my own person. I took six grains every three hours until I had taken all of the eighteen grains which had been left with me. Except a slight sense of fullness in the head, I had none of the usual sensations which accompany the other salts of bark, no nervous tremulousness and no tinnitus aurium. Although the quantity taken seemed hardly sufficient to prevent a return of the paroxysm, I have never had a return.

Thc results of this experiment on myself seemed to justify my use of it with my patients. I have kept a record of seventeen cases in which I have used it, and in all but one with perfect success. In this case the patient knew that it was a substitute for the sulphate of quinine which I was using, and as none of the usual phenomena which follow its administration occurred, he lost faith, and substituted some pills of quinine which he had in the house.

As there is such a monotony in the report of cases of malarial fever, I spare your readers a formal report of each case, one very like the other, with two exceptions. One, a case in which the sulphate of quinine invariably induced urticaria of such severity as to almost deter the patient from taking it. In this case some urticaria did appear, but nothing to compare in severity with that which followed the sulphate. The other was a case of double quotidian, which only yielded after using pretty heroic doses.

Although this is not a sufficient number of cases to make it appear that we can discard our old and tried remedy, the sulphate, the results were so certain and unvarying as to convince me that in the dextro-quinine we have a most valuable addition to our armamentarium. It strikes me that the absence of the disagreeable effects of quinine is a great desideratum, which, with its cheapness, should recommend it to the profession. The cases in which I used it were all of simple, uncomplicated malarial fever, and do not furnish evidence that it possesses the antipyretic power which the sulphate of quinine exercises in typhoid fever, in pneumonia, and the zymotic diseases. I feel justified, by the similarity of its effects to quinine in the cases in which I used it, to feel some degree of certainty that it will not disappoint us in all diseases in which the sulphate of quinine is used. After further use I will report the results, and hope that the profession will give it a fair trial. I also hope that the experience of others will be given through the *News*, so that by a comparison of views we may find the true value of this new salt. I would state that with adults the usual form of administration was in pills, while with children I usually gave it suspended in the compound elixir of liquorice. The dose was usually the same as that of quinine.—*Louisville Med. News.*

---

**DEXTRO-QUININE** sent by mail, at the rate of $1.60 per oz., to Physicians who cannot be supplied by their local druggists. Address

KEASBEY & MATTISON, Philadelphia.

**Cholera Infantum.**—"In a discussion on cholera infantum in Academy of Sciences, Kentucky, Dr. Holloway said: In colliquative discharges I believe the camphor julep, made of camph. carb. magnesia and water, given in ice, is the best single remedy. I think highly of Dr. Polk's views of quinine; would give it by enema, and have the anus closed by the hand with a napkin until absorption of the quinine takes place. Would use opiates, and recommend whisky as a stimulant."

Dr. Clemens, said "the fatality of cholera infantum is fearfully great. It produces nearly as many deaths, in the aggregate, as consumption. I believe it to be a disease of nervous exhaustion, anæmia of the brain being due to loss of serum from the blood. When called to see cases of this disease after the stage of collapse I have the patient sponged with whisky, as well as the internal use of stimulants. Three drops of creosote to an ounce each of lime water and mint water, in teaspoonful doses every two hours, with powdered ice kept to the lips, seems often to check the disease in some of its worst stages. When vomiting is persistent and frequent I usually give one-sixteenth or one-twentieth grain of calomel until the discharges are changed. I then give quinine in the form of tannate—one grain of tannin to five of quiuine, in syrup of tolu. I give this just as soon as the stomach will tolerate it. Attempts to nourish acute cases within the first twenty-fours should not be carried beyond egg-water. I like the effects of belladonna in certain forms. In stages of collapse I think with Holloway that it is very similar to cholera in the adult. I often examined and found greatly diminished renal secretion, and suppression in some cases. I do not believe hyperæmia of the brain may be found even in the convulsive forms."

Dr. Larrabee was pleased to note the unanimity of opinion as to the pathology of this disease, agrees with Holloway and Polk in believing it to prevail quite extensively in Europe, under another name. He thinks heat is a prime factor, by its interference with the circulation in the spinal cord. Does not believe in malarial causes of it. Moist and warm atmosphere interferes with the secretions and irritates the delicately organized nervous system. The disease being essentially a nervous affection in its origin, the want of fixed pathological states makes it impossible to fix upon established rules of treatment. Thinks cool air, cold applied to the spine, of the greatest value. This disease is essentially the same as cholera in adults, the rapid collapse being due to the loss of serum. Anæmia of the brain is associated with heat, full sinuses, full veins, and anæmic arteries. The brain is not supported by the normal amount of pressure, and the rapid drainage soon exhausts the cerebral function. He thinks hypodermic morphia in cholera morbus should suggest its use in cholera infantum; thinks we ought to use it. Loss of serum demands the rapid introduction of fluids, and thinks the white of an egg in sweetened ice water a sovereign remedy. He does not think opium properly indicated in engorged states of the venous system. He thinks calomel the one great drug to be given in this disease. He gives it in 1/6 grain doses every four or five minutes until the character of the discharge is changed. Regards the camphor julep with high favor, and speaks highly of camphor water as a substitute when the julep is not at hand. Uses belladonna in cases of collapse, it produces hyperæmia of skin, where great pallor has existed. He proposes to apply cold to the spine by means of a rubber coil through which ice water is to be passed continually; it acts by reducing temperature. Does not believe the disease is peculiar to cities, though it is more likely to prevail in cities than in the country.—*Medical Herald.*

**Prophylaxis in Yellow Fever.**—Dr. Summers, in Nashville Medical Journal, says: The great problem for the medical profession to solve is the arrest of germ development in the organism. This is the only method of prophylaxis that is at all rational and likely to be followed by practical results. This being accomplished yellow fever will be robbed of its terrors, and be remanded back to that class of diseases which modern science has made controllable by the efficiency of its therapeutic agents.

Let us then see if we cannot arrive at some philosophic plan to accomplish this result.

It is well established that fermentation is the prime pathological factor in this disease. As a result of active fermentation we have the solution of a gas called carbonic acid gas. This is a solvent of albumen. In the blood albumen is not normally in a state of solution but of molecular suspension. The carbonic acid set free in this fermentation dissolves this suspended albumen, which now becomes subject to osmosis, passing out of the walls of the capillaries which in a state of molecular suspension it could not do. Carrying with it white blood corpuscles into the tissues without it sets up the process of calorification which is so remarkably characteristic of this fever. Getting into the tubes of the kidneys it coagulates to form tube casts and, if not arrested, will go on to completc clogging of the secretory apparatus. The other changes which occur in the organism during the course of the fever are those of degeneration resulting from the causes just referred to. Now let us begin at the first pathological manifestation—the fermentation set up in the blood by the proliferation of the germ. What will prevent this or arrest it when once set up? The latter is more difficult to accomplish than the former. It is a well known chemical fact that sulphurous acid gas will at once stop fermentation when brought in contact with a fermenting substance—either alone or in its combinations. It is reasonable to suppose that this could be introduced into the organism to the arrest of fermentation and so has experience proved. The hyposulphite of soda is the best preparation to use. Suppose, however, that the fermentation has gone on to such an extent as to begin the solution of the albumen of the blood, which is often the case even when the system has been saturated with sulphuric acid—what then? Well, as a matter of course, we must find something which will precipitate the albumen from its solution. Chemistry comes to our aid with phosphoric acid which will effect this promptly, either alone or in combination. So we should expect *a priori*, and have found experimentally, that phosphate of ammonia will accomplish this result.

We have here the main avenues to the pathological arena of the disease therapeutically guarded. I have found that during the late epidemic that those persons who, with proper care of their person, took daily the prescription which follows either escaped the disease or at least suffered from very mild attacks :

| | | |
|---|---|---|
| ℞ | Sodæ hyposulphite.......................... | one ounce. |
| | Ammon. Phosphat........................... | one ounce. |
| | Aq. Aurant. flor.............................. | six ounces. |

Dose, a teaspoonful in a little water, three times a day.

A little brandy every morning before breakfast, a good heart and easy mind, moderation in work, regular hours and attention to general hygienic conditions, along with the above prescription which meets the pathological conditions in advance, furnish the best basis of protection I think possible to offer, and I am satisfied that such a rational prophylaxis systematically carried out will go very far toward diminishing the horror of this disease with which its late fearful ravages have filled the mind of the people.—*Journal of Medicine and Surgery.*

**On the Injection of Warm Water into the Vagina, in Certain Cases of Labor.**—The cases to which this treatment is specially applicable are those in which the os uteri is thin and rigid and the perineum unyielding.

CASE I.—Mrs. A.——; third confinement. I was first called in about seven o'clock a.m., and found the os open just sufficiently to admit the forefinger. Pains every ten minutes. At 3.30 p.m., the os was about the size of a shilling, and the perineum rigid. Pains every two minutes, prolonged. The nurse, under my direction, administered an injection of warm water for about ten minutes, and after another ten minutes I made a vaginal examination, and found the os nearly dilated to the full and the perineum relaxed. The child was born within forty minutes from the commencement of the injection.

CASE 2.—Mrs. B——; sixth confinement. She informed me that in all her previous confinements she was in violent pains from thirty-six to forty-eight hours, but was always quick at the end. About 3 a.m. I found her with pains every two or three minutes, and the os about the size of a shilling and unyielding. Pelvis roomy and no difficulty likely to be presented after dilatation of the os. At 8.30 a.m. I was called again, and could not detect the slightest alteration in affairs; so I ordered an injection of warm water to be given her. Half an hour after the injection was administered I was sent for again, but the child was born before I could arrive, although the distance was only two minutes' walk.

CASE 3.—Mrs. C——; second confinement. About 2.30 a.m. I was called, and found the size of the os to be about that of a shilling. Pains every five minutes. At 10 a.m. there seemed to be no change except that the pains were more frequent and longer. She was ordered a warm water injection. At 12.30 p.m. I found that the nurse had not administered the injection, and that the os was very slightly enlarged. The perineum was not at all rigid. I then gave the injection myself for about five minutes, after which the os dilated very quickly. The child was born in forty minutes' time.

CASE 4.—Mrs. D——; first confinement. I was called about 10 a.m., and found the size of the os just large enough to admit the finger. The vagina and perineum were excessively rigid—in fact the most rigid I ever came across. At 12 noon the next day the os was about the size of a shilling, and the other parts were unaltered. I injected some warm water for about eight minutes, after which the os rapidly dilated, dilatation being accomplished within half an hour, and at the same time the vagina and perineum became relaxed. As the child was a very large one, it took nearly three hours to traverse the pelvis, but the last part of the labor was quickly and easily performed.

CASE 5.—Mrs. E——, aged 37 years; first confinement. At 10.15 p.m. I found the os sufficiently dilated to admit the finger, and seemingly very unyielding. Pelvis fairly roomy, but the vagina and perineum rigid. She was ordered to have an injection for twenty minutes, which was commenced at about 11.15 p.m. A few minutes past one I was again sent for, and arrived just as the child was coming into the world.

CASE 6.—Mrs. F.——; first confinement. At 11.45 I found that the os would hardly admit the forefinger, although the pains had been continuing regularly since 9 a.m. She took then a draught consisting of forty minims of tincture of opium, which, however, had no soothing effect. At 9 a.m. (twenty-four hours from the commencement of labor), the size of the os was that of a shilling. Ordered an injection of warm water. At 5 p.m. the size was hardly that of a florin. At 10 p.m. the os had remained stationary in size and extremely rigid, although the pains were almost incessant. Upon questioning the nurse, I found that only lukewarm water had been used for the injection, and thereupon I administered one myself for five minutes with water as hot as she was able to bear. After this each pain made such a great progress that the os was wholly dilated in fifteen minutes, and the child born exactly fifty minutes after the commencement of the administration of the injection.—*Lancet.*

**Extraction of Cataract.**—Mr. Charles Higgens, in Royal Medical and Chirurgical Society, read a paper entitled "Remarks on 150 Operations for Extraction of Cataract." The paper was accompanied by a printed table, in which were set down the sex and age of the patient, right or left eye, form of cataract, kind of operation, results and remarks. The results were collected under three heads—successful, partially successful and failures. Under the first head were 115, 76-6 per cent.; under the second 24, 16 per cent.; under the third 11, 7-3 per cent. So that in 92-6 per cent. of the cases the sight was improved by the operation. In 7-3 no improvement took place, or sight was worse than before. Three methods of operating were described—extraction by small flap, by linear section, by oblique corneal section. 104 cataracts were extracted by the first method, 26 by the second, and 21 by the third. The advantages and disadvantages of the various methods were briefly alluded to. Iridectomy at the time of extraction, or as a preliminary some time previously, was strongly recommended. The relative advantages of upward and downward section were mentioned. The after-treatment was given.

Mr. Macuamara said that the prognosis of cases of cataract operation was favorable in proportion to the dilatability of the pupil by atropine. He had never placed much reliance upon iridectomy in cataract extraction, but his great aim was to remove the entire lens, making a corneal section of just sufficient size to admit of this. If any fragments of the lens are left behind they set up irritation. The disadvantage of iridectomy lay chiefly in the fact that it admitted too much light into the eye, so that vision for distant objects became blurred, whilst the most successful cases he had seen had been cases of the old flap extraction and central pupil. He had lately seen a lady in whom thirty years ago Mr. Guthrie had removed cataract from both eyes; she has perfect vision.

Mr. Higgens' statistics were of value, and would advance this branch of surgery—different from figures lately published by an eminent continental oculist, of 230 cases of cataract and no failures! It is notorious how figures can be misread.

Mr. Hulke commented on the number of cases in the table of young persons, a few even at three and five years, and many under twenty years. He would like to know the reason why the author had departed from the generally accepted practice of treating such cases, where the lens is soft, by some of the various methods of solution, assisted by suction.

Mr. Spencer Watson referred to a method, introduced by a continental surgeon, whereby the capsule is lacerated before the corneal section is made. In lacerating the capsule after such section, there is greater risk of displacing the lens into the vitreous, or of causing prolapse of the vitreous. He had performed the new method some twenty-four times, and with good result; and in some cases he had found it well to perform a preliminary iridectomy. He thought the upward section of the cornea often better than the downward. It was very important to use as few instruments as possible, and to avoid the scoop.

Mr. Higgens, in reply, said that removal of the whole lens was more easily effected with a small iridectomy than without. The old flap section, in favorable cases, was very good; no doubt it was so successful in past times because no cataracts were removed until they were "ripe." He fully admitted that several of his patients were young, and that his operations were often experimental. Needle operations were tedious and dangerous.—*Lancet.*

**Cases of Syphilis.**—The following interesting cases are reported in the *Lancet*, by Dr. Jonathan Hutchinson :

A young man was admitted six weeks ago, with a large indurated sore on his lip. Its characters were most definite, and under the jaw were a number of enlarged glands. The skin was covered with a lichenoid and papular rash. He did not know how he had caught the sore. He first observed a little crack, then a month later it began to inflame and harden, and in another month the rash began to appear. Having remarked on the case as an example of non-venereal syphilis (*syphilis sine coitu*), or probably chancre from kissing, I drew attention to it as an illustration of the early stages of the disease.

For practical purposes we may divide the early phenomena of syphilis into three periods of one month each. The first month is occupied by the incubation of the chancre, and during it there is either no sore at all, or a sore which is soft, suppurating, and without definite character. At the end of the first month the process of induration will be commenced. Another month may be allowed for the full development of the chancre and for first appearance of constitutional symptoms; and at the end of two months from the contagion the sore may be expected to be hard, the bubo well characterized; the skin mottled with a roseolous rash. If treatment be avoided, the rash will develop further during another month, and, at the end of three, the chancre will still be in full vigor and the skin covered with a multiform eruption. The disease will now be at its full height, and it is at this period that iritis and retinitis are more likely to occur.

In this case the lad has now been one month under treatment by two-grain doses of gray powder three times a day; and, without ptyalism and without any interference with general health, the induration has melted away, and the rash almost wholly disappeared.

In connection with the above, in which syphilis is being cured by small doses of mercury administered through a long period, salivation being avoided, I drew attention to another, which occurred a month ago, in which profuse salivation appeared to be productive of the best results. A married woman of middle age was admitted with a most copious scaly and papular eruption. It covered her face, trunk and extremities, not any part escaping, and the profuse production of scale crusts was a very remarkable feature. On the arm it might have been mistaken for common psoriasis, but on the face there could be no doubt as to the diagnosis. Mercurial baths were ordered, but after the third she became freely salivated. For a week or two she was confined to bed, and in a very feeble state, from the profuse ptyalism. During this period the eruption vanished as if by magic. No more mercury was used in any form, and at the end of a month the woman left the hospital covered with stains, but having nothing whatever remaining but stains. She was charged to return in a month, that we might know if any relapse occurred.

I remarked in connection with this case that it was the most rapid cure of a severe secondary eruption which I had ever known. Although on the whole I prefer the long continued treatment by small doses, yet it is well to bear in mind that in certain cases mercury appears to be far more efficient when pushed to its full physiological influence. We must avoid prejudging the question, and keep our minds open for the reception of all evidence that may be forthcoming. In some acute inflammatory affections, not syphilitic, benefit is observed immediately that salivation occurs, while none is witnessed before.—*Med. Rep.*

**Oxalate of Cerium in Whooping-Cough.**—CASE 1.—A girl aged seven years. After a preliminary bronchitis the characteristic whoop declared itself. Bromide of ammonium, belladonna, chloral hydrate, etc., were given, but seemed to exert little influence, when suddenly the cough ceased, only to give way to typhoid fever, which, with its relapse, lasted for six weeks. Hardly was the patient up again before the whooping-cough reappeared, with even more severity. Expectorants were given, and one dose of 2 grs. of oxalate of cerium half an hour before breakfast each morning. The salutary effect of the oxalate was quickly apparent; but to see if its action was not overestimated, it was withdrawn for several mornings, when, instead of having light attacks of coughing once or twice a day, with nocturnal quietude, greater intensity and frequency of the attacks quickly ensued. The cerium was again resorted to, and the child soon recovered with but slight inconvenience.

CASE 2.—Girl 4 years old. Had whooping-cough two weeks, when scarlet fever appeared in a very severe form, being complicated with abscesses, uremic convulsions, etc. The pertussis did not at any time abate, but steadily increased in severity. Of course, with such a number of troubles, much attention could not be bestowed on the cough; but after convalescence as regarded the scarlatina had been established, the pertussis was treated. Oxalate of cerium was given as above, in one-grain doses, once a day before breakfast, with quick amelioration of the harassing cough. It may be here stated that, to reduce the high temperature of the scarlet fever, quinia was repeatedly given in large doses without having the least effect in counteracting the pertussis.

CASES 3 and 4.—Boy 19 months old and girl aged 3 years, attacked with whooping-cough; coughing spells frequent and severe, occurring once to three times each hour. Bromide of ammonium and belladonna were followed by no improvement, excepting easier expectoration. Epistaxis and frequent vomiting, accompanied by a cyanotic countenance, in both cases. The oxalate of cerium was given before breakfast, in one-half and one-grain doses respectively, once a day, with almost instant relief, the attacks being reduced to two or three light ones in twenty-four hours, instead of occurring hourly and with greater severity. To demonstrate the usefulness of the remedy it was withdrawn for several days, though the bromide and belladonna were continued, with the same result as in case No. 1.

The other remaining cases were uncomplicated ones, and yielded to the oxalate of cerium most satisfactorily, being relieved with the same promptness as the preceding ones. The rule was also observed in each case to continue the remedy one week longer than there was any existence of the whoop, to obviate the possibility of a relapse.—DR. MORJE, in *N. Y. Medical Record.*

**Rhus Poisoning.**—The request of Dr. D. M. Johnson for suggestions for the treatment of the poisoning by rhus toxicodendron has elicited responses from practitioners in various parts of the country. In addition to the articles on the subject by Drs. Gundrum, of Iona, Mich., and Parsons, of Brownsville, Missouri, we have received numerous others. A number of these are but recommendations of applications very commonly resorted to, and need not, therefore, be reproduced here. The following, however, are somewhat different. Dr. J. C. Campbell, of Albany, Vermont, has found satisfactory results to follow a free bathing with a saturated solution of the chlorate of potassium and the internal administration of:

℞ Tinc. ferri chloridi.................................. ʒ ij.

Cinchonidiæ sulph.................................. ʒ ss.

Aq. font. q. s. ad.................................. ℥ ij.

Dose, a teaspoonful every four hours.

Dr. J. F. Campbell, of Williamston, Mich., says the following has never failed in his hands :

℞ Tinc. camphoræ

Aq. ammoniæ..................................aa ℥ ss

Ol. Olivæ.................................. ℥ ijss

Apply three times a day. Make it stronger or weaker *pro re rata.*

Dr. H. H. Bordener, of McClure City, Pa., pins his faith strongly to the black wash of the U. S. D. He gives also internally quinine and iron.

Dr. B. C. McPhail, of Minnesota, believes that the irritation is caused by an acid which is generated by the poison, and he applies a saturated solution of the bicarbonate of soda as a chemical antidote. Whether his theory is correct or not, he has found the application better than any other he has tried.

Dr. E. T. Wilmot, of Great Bend, Pa., writes :

"In my practice, the application of tinc. iodine has been the most effectual. From one to three applications, covering the affected part completely with the liquid, has never disappointed me of a cure. The burning and smarting sensation attending such an application is more or less severe, according to the nervous susceptibility of the patient, yet they will invariably pronounce the remedy as not half as bad as the disease."

Dr. A F. Henry, of Alamo, Indiana, sends the following :

℞ Caustic potassæ.................................. gr.xl.

Rain water.................................. ℥ iv. M

Wash the parts four or five times a day, and a cure is effected in two days. If applied immediately *instant* cure results.

One of my patients, very susceptible to the poison, carries a solution in his pocket when re-setting fences, and applies it at once when exposed to the poison. Instantly the epidermis peels as after other treatment. Olive oil for after treatment.—*Michigan Medical News.*

**Baptisia Tinctoria in Typhoid Fever.**—A paper read by Dr. Laurence Johnson, of New York, at the recent meeting of the Medical Society of the State of New York, on the "Action of Baptisia Tinctoria in Typhoid Fever," is summed up as follows by the reporter for the Boston Medical and Surgical Journal: Baptisia tinctoria, although formerly believed to possess antiseptic powers rendering it useful in diseases having a tendency to putrescence, has of late fallen into utter neglect with the regular profession. It is, however, extensively employed by the eclectics and homeopaths; the latter especially considering it very valuable in typhoid fever. It was to test its value in this disease that the experiments recorded were made. The preparation used was a tincture of the root, and it was administered in small doses (from one to five drops) at intervals of from one to three hours. The ordinary general measures of treatment, such as cool sponging, milk diet and stimulants, when required, were not neglected, and any casual indications were promptly met and appropriately treated. Careful records of pulse and temperature were kept, and were shown in tables accompanying the paper.

Of the seven cases whose histories were detailed, three at least had, at the beginning, symptoms betokening attacks of severity. The pulse, temperature and general condition of these patients left no room for doubt upon the point, yet the improvement under treatment was remarkable. In general, it may be stated that the cases, when treatment was well under way, were characterized by lack of symptoms sufficiently grave to occasion anxiety. There was a marked absence of delirium and comparatively little diarrhœa, though at the beginning, in two or three cases, these were very troublesome features. The temperature seemed to be reduced by the drug, and in one case, where the morning temperature was as high as 106° F., when baptisia was employed for the first time, it never reached that point again, while the patient was fully convalescent in ten days. All the patients made good and comparatively quick recoveries.—*Exchange.*

**Phlyctenular Ulcers of the Cornea.**—Professor Seely says: "I here show you two ulcers of the cornea, near the periphery, apparently ready twenty-four hours ago to perforate. I then ordered a solution of eserine to be dropped into the eye four or five times before their return, and you never would suspect that there had ever been any danger of a perforation. I have had the most fortunate and satisfactory results with ulcers of the cornea under the influence of the myotic, and am now using it in all cases where there is no iritis. If the iris is inflamed, a mydriatic wash is to be used often enough to prevent synechia posterior.

"For corneal affections with iritis, I prefer duboisine, as during my five or six months' experience with the new mydriatic I have met with no case where it caused any irritation. I wish to call your attention especially to the use of the myotic in corneal affections of all sorts. You have seen a large number of cases of 'pannus' treated with the myotic in the most satisfactory manner. In fact, trachoma, with corneal complications, is robbed of its discouraging features by eserine and yellow oxide in vaseline eserine iritis. I have never yet met with a case of eserine iritis started up by eserine, though attention has been called to such a result; still, it is well enough to be on the lookout for such an accident.

"*Rupture of the Cornea and Prolapse of the Iris, Peripheric.*—The next case I present is one of very great interest, especially to the general practitioner.

"In general, the treatment of prolapse of the iris is, if it bulges and contains aqueous humor, to puncture it, thus causing the tumor to collapse, and then to lift it up—the prolapsed portion—with a forceps and strip it off. If the prolapse is seen early, and the iris is not lacerated, it is wise to attempt its return, under strong applications of eserine and a small probe or spatula of rubber. The eserine acts most favorably in such eases. If the wound is a large one, a compressive bandage should be applied to support the ruptured cornea."—*Lancet and Clinic.*

**Typhoid Fever During Infancy.**—The following is a well marked example of a case of enteric fever in an infant aged six months.

On Sunday, February 23d, I went to Redhill to see (with Dr. Walters, of Reigate) a little boy who was suffering severely from a relapse of typhoid fever. After our consultation, Dr. Walters was so kind as to invite me to see, in the adjoining house an infant aged six months, who presented a typical example of the disease. The child manifested the ordinary symptoms, and the eruption was abundant and pathognomonic. I understood that the disease had been contracted through the food, which was partly, if not wholly, artificial.

Dr. Walters, whom I have to thank for kindly permitting me to make this reference to the case, says, in a letter with which he has favored me: "I remember seeing a case in as young a child in a former epidemic here, which was due also to contaminated water-supply. It was also characterized by a very copious eruption." Both Dr. Walters's cases recovered.

Typhoid fever in *young* infants is rare. I have myself seen it in infants on several occasions, but I do not remember ever before having observed it at as early an age as in Dr. Walters's cases. Probably the exemption of young infants is mainly attributable to their mode of feeding; that is to say, children reared solely at the breast escape (unless their mothers suffer), while those who are either wholly or partly hand-fed are exposed to it. This at least accords with my own observation. It is well to bear in mind in this connection that, very commonly, young infants—*e. g.*, those under six months—escape the invasion of the acute specific diseases, even when other children in the family suffer. It is to be feared that typhoid fever is not unfrequently overlooked in young children; "gastric fever," "infantile remittent fever," "low fever," and "slow fever" being assigned as the cause of illness.—*British Med. Journal.*

**The Short Forceps Superseded.**—Having had some little experience in the practice of midwifery, I beg to bring under notice the following method of delivery, in preference to the use of the short forceps. Take, for example, a typical case, to which the short forceps would be used. The head of the fœtus is near the outlet of the pelvis, the pains are few and of short duration; the patient is very much exhausted, and seems to have no power of expelling the fœtus. Between the intervals of the pains, the head recedes into the pelvis and returns to its former position at the return of the pains, but does not make any progress. My plan is this: At the outset of the pains, I pass the index finger of my left hand into the rectum of the patient, and, as it were, hook the head of the fœtus, so as to keep it from returning again into the pelvis, till the patient has a return of the labor-pains; and by continued pressure on the superciliary ridge of the fœtus, I gradually expel the head. This method I have adopted in several cases with marked success, and several of my friends have adopted the same method, at my suggestion, with the same favorable results—of course, always premising that the strength of the patient has been kept up by suitable stimulants; and, if thought advisable, ergot have been given to cause uterine contraction.—*Pract. Med. and Surg.*

**Quebracho, a New Remedy in Dyspnœa.**—Penzolt received, some time ago from Brazil, a package of bark from the *Apidosperma quebracho*, a tree belonging to the *apocyneæ*, said by South American physicians to possess anti-febrile properties. An alkaloid has been extracted from this by Baeyer, but this has not yet been tried, a tincture of the bark being employed by Penzolt, who found little or no anti-febrile effect from its use. On the other hand, he found it of value in various forms of dyspnœa. The formula used was as follows: Ten parts pulverized bark were extracted for a week with one hundred parts alcohol, filtered, evaporated and dissoved in twenty parts of water. Of this solution, one or two teaspoonfuls were given twice or thrice daily. It could be continued indefinitely without fear.

Penzolt concludes as follows: "We possess in quebracho bark a remedy which, without any ill effects, diminishes or puts an end, within an hour, to various forms of dyspnœa in the different diseases of the lungs and circulatory apparatus. The effect produced is manifested by a reduction in the frequency of respiration, often in the depth of the cyanosis, and, above all, in the subjective anguish.

The bark, which alone is as yet procurable, is used in commerce for tanning leather.—*Berlin Klin. Wochens.*

**Explosive Mixture.**—An explosive mixture is found by the combination of tincture of cardamon with nitro-muriatic acid (Salpetersalzsaure). That *Zeitschr. d. allg. Apoth.-Ver.* states that a druggist recently filled a prescription composed of these ingredients and handed the bottle to the messenger, in whose pocket it almost immediately exploded. The mixture was again made, the messenger warned not to shake the bottle. He arrived home safely, but in opening it the second exploslon took place, seriously injuring the patient's eyes. The druggist should have first mixed the two acids, and should not have closed the vial after the addition of the tincture until all reaction had ceased.

**Massage of the Tonsils.**—M. Quinart describes, in the Archives Medicales Belges, a method of treating hypertrophy of the tonsils that has proved very successful in his hands. The method, which is only applicable after the inflammatory period has passed, consists of massage of the gland, and is carried out as follows: He covers his index finger with alum, introduces it into the mouth, and brings it to bear directly on the tonsil, which is manipulated, with gradually increasing force, over as great an extent of its surface as can be reached. The operation is at first painful and disagreeable; but the discomfort is readily allayed by an emollient gargle. After a few repetitions, it ceases to be painful, and the patients readily learn to practice it themselves.—*New York Medical Record.*

**Discoverer of Anæsthesia.**—The National Eclectic Medical Association, at its recent session, unanimously awarded to Dr. Crawford W. Long, of Georgia, the credit of the discovery of modern surgical anæsthesia. His claims are becoming more and more established in the minds of all who are examining the matter without the bias of early education or sectional pride.

By the way, it seems that Congress has ordered the establishment of a National Gallery of Statuary in Washington, D. C. Each State is to designate two of its most famous deceased citizens, whose memories are thus to be perpetuated, It has been suggested that Georgia should contribute to this National Gallery, the statue of Dr. Crawford W. Long, late of Athens, Ga.

**The Œsophageal Sound in Hiccough.**—L'Union Medicale, March 17th, contains the report by Barre of a case of hiccough in a man aged forty-five, which had been only partially relieved by ether, opiates, bromides, compression and chloroform. This continual disturbance lasted ten months, when Barre one day passed an œsophageal sound. His patient fainted, and it was withdrawn. On the next day the patient reported that he had since the introduction of the sound had only three slight attacks. The operation was then repeated, no inconvenience resulting this time. At the date of report, thirty-seven days after, no recurrence of the hiccough had taken place.—*Cincinnati Lancet and Clinic.*

**Peach Kernels for Clearing Water.**—A Dutch hotel-keeper in the Transvaal clarifies the turbid water of the district in the following way: Half a dozen peach kernels are slightly cracked and thrown into a large butt of water. In an hour or two the muddiest will be found beautifully clear.—*New Qemedies.*

**Ergot vs. Lactation.**—Dr. Russell reports to the Bell County Medical Society a case where the secretion of milk was entirely prevented by the use of ergot until the eighth day. Ten hours after the medicine was discontinued the secretion of milk was quite free, but was at once checked by the use of ergot again.—*Nat. Med. Review.*

**Apiol in Amenorrhea and Dysmenorrhea.**—Apiol, the active principle of parsley, is an oleaginous amber colored liquid, soluble in water in any proportion, and of an acrid taste. It may be given in capsules. According to Marotte, in Mouvement Medical, it brings on menses, regulates menstruation, and calms the pains by which it is often accompanied. It has no action upon the pregnant womb.

**Facetiæ.**—Devil's Drug was one of the old names for assafetida, *a propos* of which the Medical Press tells the following: A small boy, entering the shop of a Scotch druggist, said—"My mither sent me for a bawbee's worth of that stuff for the wind. She disna' mind the right name, but they ca't deevil's dirt." To which the northern knight of the pestle replied—"Rin hame and tell your mither that I canna' disturb the devil for less than a penny."

"GOD and the doctor we alike adore,
But only when in danger—not before;
The danger o'er, both are alike requited—
God's forgotten, and the doctor slighted."

—*Southern Clinic.*

